# Listeriosis associated with pre-prepared sandwich consumption in hospital in England, 2017

**DOI:** 10.1017/S0950268821001916

**Published:** 2021-09-30

**Authors:** J. McLauchlin, H. Aird, C. Amar, G. Boyd, A. Brindle, T. Dallman, K. Jalava, A. Painset, A. Simbo, M. Swindlehurst

**Affiliations:** 1 Public Health England Food Water and Environmental Microbiology Services, National Infection Service, Colindale, London, NW9 5EQ, UK; 2 Public Health England Food Water and Environmental Microbiology Laboratory York, National Infection Service, York Biotech Campus, York, YO41 1LZ, UK; 3 Public Health England Gastrointestinal Bacteria Reference Unit, National Infection Service, 61 Colindale Avenue, London, NW9 5EQ, UK; 4 Department of Pathology, Calderdale & Huddersfield NHS Foundation Trust, Huddersfield, West Yorkshire, HX3 0PW, UK; 5 Department of Health and Wellbeing, Environmental Health, Food Safety Team, City of Bradford Metropolitan District Council, 5th Floor, Britannia House, Broadway, Bradford, BD1 1HX, UK; 6 Emerging Zoonotic Infections & Travel Health Division, Public Health England Gastrointestinal Pathogens Unit, Tuberculosis, Acute Respiratory, Gastrointestinal, National Infection Service, 61 Colindale Avenue, London NW9 5EQ, UK

**Keywords:** food and environmental surveillance, food safety in hospital, *Listeria monocytogenes*, listeriosis, pre-prepared sandwiches, whole genome sequencing

## Abstract

A case of listeriosis occurred in a hospitalised patient in England in July 2017. Analysis by whole genome sequencing of the *Listeria monocytogenes* from the patient's blood culture was identified as clonal complex (CC) 121. This culture was indistinguishable to isolates from sandwiches, salads and the maufacturing environment of Company X which supplied these products widely to the National Health Service. Whilst an inpatient, the case was served sandwiches produced by this company on 12 occasions. No other cases infected by this type were detected in the UK between 2016 and 2020. Between 2016 and 2020, more than 3000 samples of food, food ingredients and environmental swabs from this company were tested. *Listeria monocytogenes* contamination rates declined after July 2017 from 31% to 0.3% for salads and 3% to 0% for sandwiches. A monophyletic group of 127 *L. monocytogenes* CC121 isolates was recovered during 2016–2019 and was used to estimate the time of the most recent common ancestor as 2014 (95% CI of between 2012 and 2016). These results represent persistent contamination of equipment, food contact surfaces and foods at a food manufacturer by a single *L. monocytogenes* strain. Colonisation and persistent contamination of food and production environments are risks for public health.

## Introduction

Listeriosis is a rare, serious systemic infection caused by the bacterium *Listeria monocytogenes.* The disease is predominantly a foodborne illness and most often affecting those over 60 years of age, the immunocompromised as well as pregnant women with their unborn or new-born infants [1]. Listeriosis is the most severe foodborne infection reported in the European Union in terms of death and hospitalisation [2]. Cases occur following consumption of contaminated food and are predominantly sporadic or part of small clusters, but occasionally occur as large outbreaks [1, 3]. Contamination of foods associated with listeriosis can be at primary production, or more frequently, originate from food production environments where the bacterium can colonise harbourage sites for years and even decades [4]. The disease has a low attack rate and variable (1–90 day) incubation period [5, 6]. Because of these features together with highly complex food supply chains, there are challenges in linking specific foods to infection, and the proportion of all cases where specific food exposures are identified is small [3], even with the unprecedented discriminatory power of whole genome sequencing (WGS [7]). Therefore, it remains important to consolidate data from cases of listeriosis to provide a better understanding of the most appropriate interventions to control this disease.

The annual totals of human listeriosis reported in the UK varied between 160 and 201 cases per year between 2015 and 2019 (0.23–0.31 cases per 100 000 population [2]). Public Health England (PHE) coordinates surveillance of listeriosis in England and Wales [8], and also performs testing of food, water and environmental samples in England. Data originating from epidemiological, clinical and food/environmental investigations are combined with the results from WGS to enable the detection of associations and elucidate the sources of contamination [8, 9]. In December 2015, PHE introduced WGS as a routine service for the characterisation of all referred isolates of *L. monocytogenes* for public health purposes with selected isolates analysed prior to 2015.

The consumption of pre-prepared sandwiches served in hospitals was the most common food vehicle identified for human listeriosis for England and Wales between 1981 and 2015 [3]. Data on 11 incidents of listeriosis associated with pre-prepared sandwiches served in hospitals occurring between 1999 and 2014 have been previously reported [3, 9, 10], and, in addition to the cases reported here, an outbreak of nine cases associated with chicken sandwiches served in hospitals occurred in 2019 [11].

In July 2017, a case of listeriosis in a 53-year-old patient in a hospital in Yorkshire and Humber Region, England, was reported to PHE [12]. Analysis by WGS of the *L. monocytogenes* from the blood of this patient was shown to be less than five single nucleotide polymorphisms (SNPs), i.e. genetically indistinguishable, to isolates from sandwiches and salads produced by Company X, which was also located at a single site in the Yorkshire and Humber Region. Company X was an approved food manufacturer producing sandwiches and salads to a wide range of premises including the National Health Service throughout England. The purpose of this study was to summarise information on the investigation of the case of listeriosis as well as microbiological monitoring of relevant food chains between 2016 and 2020.

## Materials and methods

### Case definition

A case of listeriosis was defined as a person with an illness clinically compatible with a diagnosis of listeriosis and with the isolation of *L. monocytogenes*, usually from a normally sterile anatomical site. Case ascertainment and food history were collated as previously described [8]. The listeriosis case described here which occurred in July 2017 was treated in hospitals 1 and 2 in the Yorkshire and Humber Region and all food samples collected from hospitals were also located within this region.

### Food and environmental samples

Food and environmental samples were collected by local authority sampling officers and submitted to PHE Food Water and Environmental (FW&E) microbiology laboratories located in York as part of official control activities. As part of the due diligence by hospitals 3 and 4, routine monitoring of foods (including sandwiches) was also carried out and testing was performed by the same laboratory. Samples were also tested by this laboratory under commercial contracts for the National Health Service and for the food manufacturer.

In this investigation, food samples (usually of at least 100 g) as well as environmental surface swabs and other environmental samples were collected either by local authority sampling officers, staff in the PHE FW&E laboratory, hospital catering or by the sandwich manufacturer. Samples were transported in accordance with the Food Standards Agency Food Law Code of Practice [13].

A total of 2460 samples of salad (1104) or sandwiches (1356) were collected from hospitals 3 and 4. Three hundred and seventy-one of these were collected between January 2016 and June 2017 (153 salads and 218 sandwiches) and 2089 samples were collected between July 2017 and the end of 2019 (951 salads and 1138 sandwiches). A total of 743 salads, sandwiches, food ingredients or environmental samples were collected at the premises of Company X. The samples from the manufacturer comprised 11 sandwiches collected between January 2016 and June 2017 and the remainder, between July 2017 and the end of 2020, and comprised 13 salads, 248 sandwiches, 163 food ingredients and 308 environmental swabs or water samples.

### Microbiological testing

A 10^−1^ homogenate of each food sample was prepared in Buffered Peptone Water (BPW), according to ISO 6887-1:2017 [14], which was used to enumerate *Listeria* species (including *L. monocytogenes*) (based on ISO 11290-2:1998/Amendment 1:2004 but with the variation that 0.5 ml of sample homogenate was inoculated onto a single agar plate [15]). A 25 g portion of each sample was also tested for the presence of *Listeria* species using an enrichment procedure (ISO 11290-1:1996/Amendment 1:2004 [16]). Swabs were immersed in 100 ml of half-strength Fraser broth and tested similarly to that above [16]. Identification of *Listeria* isolates was performed as outlined in the standard methods above.

#### Characterisation of *Listeria monocytogenes* isolates by WGS

Cultures of *L. monocytogenes* (one per food or environmental sample) were sent to the PHE Gastrointestinal Bacteria Reference Unit (GBRU) for confirmation and further typing: all *L. monocytogenes* were subjected to WGS [17, 18]. DNA from purified cultures of *L. monocytogenes* was obtained by automated extraction (QIAsymphony DSP DNA Kit, Qiagen, Manchester, England) according to manufacturer instructions. Genomic DNA was sequenced by the PHE National Infection Service Central Sequencing Laboratory: sample preparation was using the NexteraXT (Illumina Inc, San Diego, USA) and sequenced using Illumina HiSeq 2500 platform with 2 × 100 bp reads (Illumina Inc). Short reads were quality trimmed using Trimmomatic removing the sequence adaptor [19]. Clonal complexes (CCs) were derived from WGS analysis and were assigned using MOST [20] in accordance with the designation of the Institut Pasteur international MLST database for *L. monocytogenes* (http://bigsdb.pasteur.fr/listeria/listeria.html). Pairwise SNP distances were calculated within CCs, then single linkage clustering was applied to derive a seven-digit threshold SNP address [17]. Isolates linked within a five SNP single linkage cluster were considered to be part of the same point source with each culture having ≤5 SNPs difference with at least one other culture within that same cluster, together with epidemiological evidence. A core SNP alignment for isolates within a cluster was generated using SnapperDB [17], recombination removed using Gubbins [21]. A Maximum-likelihood phylogeny was produced from the SNP alignment using RAxML v8.2.8 [22] under the GTRCAT model to confirm the five-SNP clustering was monophyletic.

WGS data are maintained in a PHE curated database named the Gastro-data Warehouse (GDW). At the time of completing this analysis (March 2021), GDW contained over 5400 sequences derived from *L. monocytogenes* cultured from clinical cases of listeriosis, food and the environment, which were recovered in the UK between 2009 and August 2020. Sequence reference numbers from the cultures described in this study are deposited to the Short Read Archive (BioProject PRJNA248549). Sequence reference numbers appear in supporting data (Supplementary Table S1). Data were extracted from the GDW onto Excel spreadsheets and combined with metadata associated with the food and environmental isolates.

Timed phylogeny was reconstructed using BEAST–Markov chain Monte Carlo (MCMC) v1.8.2 [23]. An alternative clock model adapted from elsewhere [24, 25], and population priors were computed, and suitability assessed based on Bayes factor tests. A model with a strict molecular clock rate under a constant population size associated with the nucleotide substitution model HKY was used and the model was run with a chain length of one billion. A maximum clade credibility tree was reconstructed by using TreeAnnotator v1.8.2 [17].

## Results

### The case of listeriosis and epidemiological investigations

A 53-year-old male with severe ulcerative colitis was admitted to hospital 1 in mid-June 2017 and discharged in early July 2017. The patient was readmitted to hospital 2 in mid-July 2017 with symptoms of confusion, diarrhoea and vomiting. *Listeria monocytogenes* was isolated from a blood culture on 18 July 2017. Analysis by WGS of the *L. monocytogenes* from this patient blood showed this to be serovar 1/2a, CC121 and less than five SNPs, i.e. genetically indistinguishable, to isolates from sandwiches collected from Company X in December 2016. A food history taken from the patient indicated that for the 30 days prior to the onset of illness he had only consumed food supplied by hospital 1, and whilst an inpatient, he was served sandwiches produced by Company X on 12 occasions.

### Examination of foods produced by Company X and inspection of the production environment before the onset of infection in the case: January 2016–June 2017

A total of 168 finished foods produced by Company X were collected from hospitals 3 and 4 in 2016 as part of their routine microbiological monitoring: *L. monocytogenes* was isolated from eight samples, other *Listeria* species from 13: all *Listeria* were detected at <20 cfu/g (Table 1). These samples comprised 102 between January and September 2016: no *L. monocytogenes* were detected, and other *Listeria* species were detected in six (6%) samples. *Listeria monocytogenes* was detected in five (28%) and other *Listeria* species from four (22%) out of 18 samples collected in October 2016. Further testing of 46 foods was carried out in November 2016: *L. monocytogenes* was detected in three (7%) and other *Listeria* species in three (7%) samples. A further 18 samples were tested in December 2016 and *Listeria monocytogenes* was detected five together with other *Listeria* species in four samples (Table 1). Following these results, the local authority was informed and visited and inspected Company X in December 2016. As part of this inspection, five sandwiches were collected: *L. monocytogenes* and *Listeria seeligeri* were detected at <20 cfu/g in each of two egg mayonnaise sandwich samples (Table 2).

**Table 1.** Detection of *Listeria* in 2460 salads and sandwiches sampled in hospitals and produced by Company X

	Number of samples tested (%)				
	2016	2017 Jan–June	2017 July–Dec	2018	2019
Salads (finished products) *n* = 1104					
*L. monocytogenes* detected	5 (10%)^a^	31 (31%)^b^	26 (11%)	11 (2%)	1 (0.3%)
*Listeria* species (not *L. monocytogenes*) detected	6 (11%)^a^	5 (5%)	25 (11%)^c^	66 (15%)	10 (4%)
*Listeria* not detected	45 (86%)	65 (64%)	179 (78%)	367 (83%)	266 (96%)
Total tested	52	101	230	444	277
Sandwiches (finished products) *n* = 1356					
*L. monocytogenes* detected	3 (3%)^d^	0	3 (1%)	5 (1%)	0
*Listeria* species (not *L. monocytogenes*) detected	7 (6%)^d^	1 (1%)	7 (3%)	21 (4%)	4 (1%)
*Listeria* not detected	106 (93%)	103 (99%)	228 (96%)	471 (95%)	399 (99%)
Total tested	114	104	238	497	403

a Four samples *L. monocytogenes* detected together with *L. innocua* (three samples) or *L. seeligeri* (one sample).

All *Listeria* detected at <20 cfu/g except for: ^b^*L. monocytogenes* was detected at 20 cfu/g in one sample of quiche lorraine salad; ^c^*L. innocua* was detected at 20 cfu/g in one sample of corned beef salad.

d One sample *L. monocytogenes* detected together with *L. innocua.*

**Table 2.** Detection of *Listeria* in 743 salads, sandwiches, food ingredients and environmental samples collected at the premises of Company X

	Number of samples tested (%)					
	2016	2017 Jan–June	2017 July–Dec	2018	2019	2020
Salads (finished products, *n* = 13)						
*L. monocytogenes* detected	0	0	2 (15%)	0	0	0
*Listeria* species (not *L. monocytogenes*) detected	0	0	3 (23%)	0	0	0
*Listeria* not detected	0	0	8 (62%)	0	0	0
Total tested	0	0	13	0	0	0
Sandwiches (finished products *n* = 259)						
*L. monocytogenes* detected	1 (20%)	0	3 (5%)	2 (2%)	0	1 (10%)
*Listeria* species (not *L. monocytogenes*) detected	1 (20%)	0	4 (7%)	15 (12%)	10 (17%)^a^	0
*Listeria* not detected	3 (60%)	6 (100%)	48 (87%)	108 (86%)	48 (83%)	9 (90%)
Total tested	5	6	55	125	58	10
Food ingredients (*n* = 163)						
*L. monocytogenes* detected	0	0	2 (8%)	2 (6%)	1 (1%)	0
*Listeria* species (not *L. monocytogenes*) detected	0	0	0	7 (21%)	6 (9%)	0
*Listeria* not detected	0	0	23 (92%)	24 (73%)	63 (90%)	35 (100%)
Total tested	0	0	25	33	70	35
Environmental samples (swabs, *n* = 306; and water, *n* = 2)						
*L. monocytogenes* detected	0	0	8 (13%)	0	2 (1%)	0
*Listeria* species (not *L. monocytogenes*) detected	0	0	0	5 (11%)	18 (11%)	0
*Listeria* not detected	0	0	55 (87%)	42 (89%)	136 (87%)	42 (100%)
Total tested	0	0	63	47	156	42

All *Listeria* detected at <20 cfu/g except for ^a^where *L. seeligeri* was detected at 40 cfu/g in a chicken mayonnaise sandwich.

Further collection of samples from hospitals 3 and 4 was performed between January and June 2017, *L. monocytogenes* was detected in 31 (31%) of 101 salads tested and none of 104 sandwiches, all at <20 cfu/g except for one sample (a quiche lorraine salad) where the bacterium was detected at 20 cfu/g (Table 1). *Listeria* species other than *L. monocytogenes* were detected in 6 (3%) of these 205 salads and sandwiches, all at <20 cfu/g (Table 1).

### Examination of foods produced by Company X and inspection of the production environment after the onset of infection in the case: July 2017 to June 2020

Following the investigation of the case of listeriosis in June 2017, further sampling and testing was carried between July 2017 and June 2020 from both Company X and hospital 3 (Tables 1 and 2). The sampling comprised 2089 finished salads and sandwiches collected in hospital 3 (Table 1), together with 732 samples (261 salads and sandwiches, 163 food ingredients and 308 environmental samples) collected directly from Company X (Table 2). *Listeria monocytogenes* contamination rates of Company X's products collected in the hospitals during the first and second half of 2017 were 31% (31/101) and 11% (26/230) for salads and 0% (0/104) and 1% (3/238) for sandwiches, respectively: all were contaminated at <20 cfu/g except for one quiche lorraine salad sample collected in the first half of 2017 where the bacterium was detected at 20 cfu/g in one sample. The subsequent contamination rates for 2018 and 2019 were 2% (11/444) and 0.4% (1/277) for salads and 1% (5/471) to 0% (0/399) for sandwiches (Table 1). *L. monocytogenes* contamination rates detected in both salads and sandwiches collected from the Hospitals as well as directly from Company X's factory declined from July 2017 after the incident and control measures were implemented (Table 2). Contamination rates for other species of *Listeria* increased between 2017 and 2018 in both foods collected at the hospitals and for sandwiches collected from Company X but declined in 2019 and 2020 (Tables 1 and 2).

### Food and environmental contamination with *Listeria*

*Listeria monocytogenes* and other *Listeria* species were recovered from products with a wide range of different ingredients (Table 3). The range of ingredients was representative of the salads and sandwiches produced by Company X: *Listeria* were not detected in other heat-treated foods (quiche, ploughman's lunch, fried fish and meat pies) produced by this manufacturer which were also supplied to hospitals (results not given).

**Table 3.** Distribution of *L. monocytogenes* and *Listeria* species (not *L. monocytogenes*) isolated from different food types or ingredients either prepared by or collected from Company X

Ingredients	Numbers of samples				
	*L. monocytogenes* detected			*Listeria* species (not *L. monocytogenes*) detected	
	Salads	Sandwiches	Other	Salads	Sandwiches
Company X					
Cheese					1
Chicken		6			24
Egg		2			1
Mayonnaise		8			16
Pork	2			4	3
Quiche					
Salmon					2
Tuna		3			2
Other			2^a^		
Total^b^	**2**	**11**	**2**	**4**	**33**
Hospitals					
Beef (including corned beef)	21			26	3
Cheese	24	1		19	7
Chicken	10	3		13	10
Duck					1
Egg		11			7
Mayonnaise		15			9
Pasta	3			1	
Pork	3			5	4
Prawn					1
Quiche	17			24	
Salad^c^					4
Salmon					1
Tuna	22	6		21	6
Turkey	2			7	1
Total^b^	**97**	**21**		**111**	**41**

a Food ingredients: lettuce and cooked sweetcorn.

b Since some foods had multiple ingredients (e.g. egg or tuna mayonnaise) the sum of samples with individual ingredients will be greater than the totals.

c Sandwiches only.

Sampling from Company X's production environment (swabs of drains and a water sample from a vegetable washer) showed contamination between July 2017 and July 2019 with the *L. monocytogenes* CC121 implicated with the infected case. A second *L. monocytogenes* strain (CC9) was detected on two occasions in August 2017 from a drain swab and the butter depositor: this strain was not recovered from any of the foods or food ingredients tested or from any other cases of listeriosis in the UK. *Listeria seeligeri* was detected in environmental swabs (drains and drain cover, utensils, a hand rail, a line belt, line sealer, cleaning brush, a vegetable slicer, floor swabs and trolley wheels) from the food preparation area between June 2018 and July 2019. *Listeria welshimeri* was detected on a drain cover, line belt and line sealer from the production area between October and December 2019.

The contamination with respect to durability data was available for 137 samples (113 salads and 24 sandwiches) collected from the hospital and where *L. monocytogenes* and/or other *Listeria* species were detected: 88 (64%) were tested 2 days before expiry, and of the remainder, two (1%) and 32 (23%) samples were tested 4 and 1 day before expiry, respectively, and 15 (11%) were tested on the day of expiry.

### Phylogenetic and time series analysis of *L. monocytogenes* and time series analysis of *Listeria* in the food chain

**Fig. 1.**
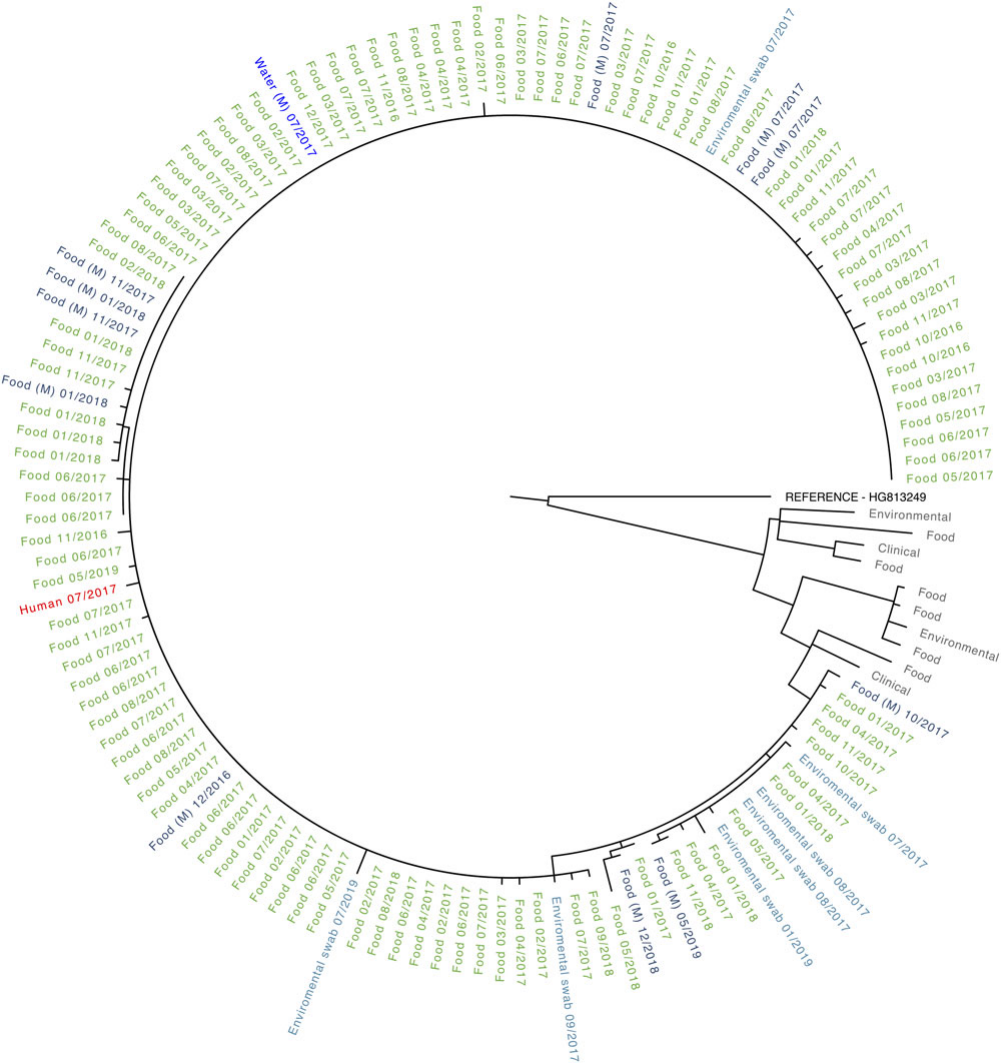
Maximum-likelihood phylogeny of monophyletic group of *L. monocytogenes* CC121 associated with Company X. Isolates were recovered from the blood culture of a case of listeriosis (red), food from hospital (green) and food from factory (labelled M, dark blue), the factory environment (light blue) or factory water (bright blue) associated with Company X. Month and year of isolation are shown for each isolate. CC121 outliers and reference (HG813249) group is shown in grey. The outliers were derived from isolates from two clinical cases, six foods and two environmental isolates examined in the UK and were <25 SNPs from the monophyletic group but with no identified relationship to the food manufacturer described here. All sequences including the outliers are available from SRA and accession numbers are listed in the Supplementary data.

Of the total of 142 *L. monocytogenes* isolates, 140 (99%) were subject to WGS. One hundred and twenty-seven of the isolates were CC121: one culture was recovered from the blood of the listeriosis patient in June 2017, the remainder were from food or the environment associated with Company X between October 2016 and July 2019 (Table 4). Sequences from all 127 CC121 isolates showed no evidence for recombination and formed a monophyletic ≤10 SNP cluster (Figure 1) with a mean SNP distance of 2.14 (maximum 14 SNPs). Retrospective analysis of the GDW database in March 2021 did not identify any *L. monocytogenes* sequences within 25 SNPs of this CC121 cluster recovered from clinical, food or environmental settings in the UK between 2009 and 2020.

**Table 4.** Quarterly distribution of 141 *L. monocytogenes* and 208 *Listeria* species isolated from 3203 food water and environmental samples associated with Company X, 2016–2020

Quarter	Total number of food, water or environmental samples collected	Numbers of specimens where *Listeria* was detected					
		*L. monocytogenes* CC 121	Other *L. monocytogenes* (CC)	Other *Listeria* species			
				*L. innocua*	*L. ivanovii*	*L. seeligeri*	*L. welshimeri*
2016							
Jan–March	33	0	0	1	0	3	1
April–June	44	0	0	0	0	0	0
July–Sept	30	0	0	0	0	0	1
Oct–Dec	64	6	3 (CC87)^a^	6	0	2	0
2017							
Jan–March	47	23	1 (CC2)^b^	0	0	0	2
April–June	166	35	1 (CC8)^b^	0	0	1	3
July–Sept	345	32	2 (CC9)^a^	0	0	16	8
Oct–Dec	277	11	1 (CC1)^c^	0	0	14	1
2018							
Jan–March	288	10	0	5	0	18	0
April–June	261	1	0	0	0	11	1
July–Sept	278	2	1 (CC18)^b^ 1 (CC1)^c^	2	2	24	0
Oct–Dec	319	2	4 (CC1)^a^	24	0	27	0
2019							
Jan–March	338	1	0	4	0	7	0
April–June	313	2	0	4	0	7	0
July–Sept	258	1	0	0	0	8	0
Oct–Dec	55	0	0	0	0	0	5
2020							
Jan–March	50	0	0	0	0	0	0
April–June	37	0	1 (CC2)^b^	0	0	0	0

CC, clonal complex.

a ≤5 SNP clusters.

b Unique SNP type.

c Two isolates belonging to CC1 which were <5 SNP apart.

**Fig. 2.**
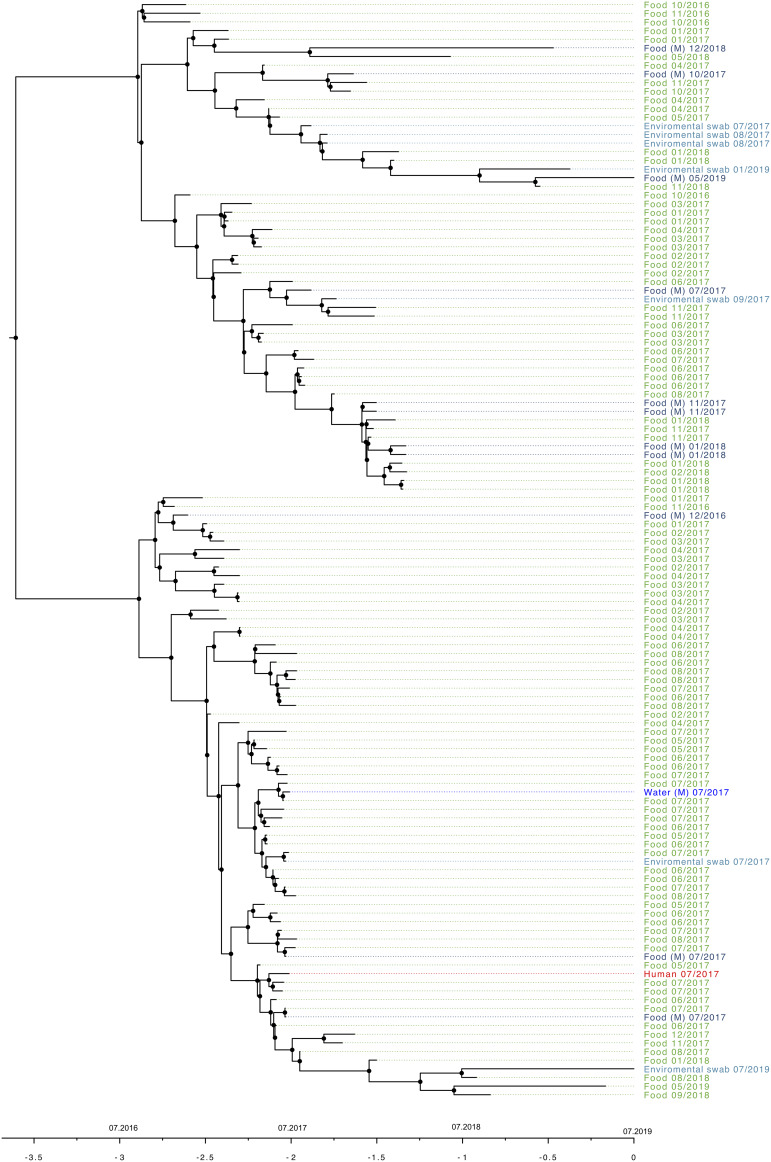
Timed phylogeny of the monophyletic group of *L. monocytogenes* CC121 associated with Company X with a strict clock and constant population size. Time is on the bottom with horizontal bars representing confidence intervals. Isolates were recovered from the blood culture of a case of listeriosis (red), food (green), the factory environment (light blue) or factory water (dark blue) associated with Company X. Month and year of isolation are shown for each isolate in this food chain.

Isolates recovered over the 3-year period were subjected to a timed phylogeny (Fig. 2) to derive the mutation rate and the age of the most recent common ancestor. The selected model with a strict clock and a constant population size gave us an estimated mutation rate of 1.29 SNPs on the genome per year. The age root was estimated to be in 2014 with a 95% confidence interval between 2012 and 2016.

In addition to *L. monocytogenes* CC121 cultures, there were a further 15 *L. monocytogenes* isolates of other types from products in this food chain which segregated into eight ≤5-SNP clusters. Four of these clusters contained one unique isolate; three were recovered from Company X's salads sampled in hospital 3 in February 2017 (CC2), June 2017 (CC8) and September 2018 (CC18), together with a CC2 isolate from a tuna mayonnaise sandwich collected from the factory in April 2020. The remaining 11 isolates were segregated into four ≤5-SNP clusters of between two and four cultures per group, three of which were all recovered over a <2-month period: i.e. three CC87 isolates from salads or a sandwich recovered from hospital 4 in October and November 2016, two CC9 isolates from the environment of Company X in August 2017, and four CC1 isolates recovered from sandwiches collected either from the factory or from hospital 3 in October and December 2018. The final cluster comprised two CC1 isolates which were recovered from a salad and a sandwich collected from hospital 3 in November 2017 and September 2018. Analysis in March 2021 did not detect any further UK *L. monocytogenes* sequences recovered between 2009 and 2020 within the GDW database which were within 25 SNPs of any of these eight SNP types except for two additional CC1 isolates which were <5 SNPs away from the salad and a sandwich isolate collected from hospital 3 in 2017 and 2018. There was no further information on these two additional isolates except that they were from food or environmental samples tested in 2016 and 2017 but were not identified as part of this food chain.

A total of 208 *Listeria* species other than *L. monocytogenes* were recovered from this food chain: 56 from samples collected directly from Company X's manufacturing premises and 152 from finished salads (*n* = 111) or sandwiches (*n* = 41) collected from hospital 3 or 4. Of the samples collected from Company X's production site, 19 were from the environment, four from salads and 33 from sandwiches. There was evidence for persistent colonisation of Company X's food production environment with other *Listeria* species over specific periods (Table 4). For example, *L. seeligeri* was recovered from Company X's production site: from the environment on 14 occasions between June 2018 and July 2019; from four salads in September and October 2017; and from 24 sandwiches between December 2016 and August 2019. *Listeria seeligeri* was also recovered from 94 foods collected directly from the hospitals 3 and 4: 68 salads between January 2016 and June 2019 and 26 sandwiches collected between April 2017 and June 2019. *Listeria seeligeri* was not recovered from any of the 142 food or environmental samples collected between October 2019 and June 2020.

### Inspections of the food production facility and corrective actions implemented

As a result of the December 2016 inspection, Company X implemented remedial actions (including reviews of cleaning) and, in accordance with the NHS Supply standards, commissioned its own microbiological swabbing and sampling on a weekly basis using a commercial UKAS accredited laboratory (not a PHE FW&E microbiology laboratory). *Listeria monocytogenes* had not been detected in any of the samples from the company sent to the commercial laboratory in the first half of 2017. The company were aware of results from testing the sandwich samples taken by the Local Authority in December 2016 but because their own testing was subsequently negative, were unaware of further issues until they were informed of the results of the samples taken directly from hospitals in June 2017.

The local Environmental Health Officers visited Company X's premises in July 2017 and August 2017 and observed that the procedures were generally of an adequate standard but that some changes to their layout to expand their production area had been recently implemented. There were concerns including sanitisation systems for vegetable washing machines, trolley wheel disinfection before moving from low to high-risk areas and shoe changing procedures. There was evidence of floor-level drainage from a low to high-risk area with a build-up of debris. During the August visit, duplicate food and environmental samples were taken and sent to the company's private laboratory as well as to the PHE FW&E laboratory: the private laboratory did not detect *L. monocytogenes* in those samples but the PHE FW&E laboratory did.

Following the results of these inspections, a commercial consultant from a cleaning company revised daily deep cleaning procedures and protocols. Company X replaced equipment and further modified cleaning procedures. The drains were cleaned daily and deep cleaned each weekend. Improvements to the floor covering were agreed to be implemented over the longer term and dry floor cleaners were used to minimise floor water. Repeated sampling of harbourage sites at the factory including where the bacterium was recovered was implemented, with all samples being tested at the PHE FW&E laboratory in York.

## Discussion

A sporadic case of listeriosis is described with evidence for exposure to *L. monocytogenes* in hospital from the consumption of contaminated sandwiches produced by Company X. The contamination of the products from this company was initially detected as a result of unrelated microbiological monitoring of food which predated the investigation of this case by 18 months. Strong evidence for a causal link between this sporadic case of listeriosis and contaminated sandwiches is provided by the consumption of Company X's sandwiches by the patient whilst in hospital and the identification of indistinguishable *L. monocytogenes* from the case's blood culture as well as from food and the food production environment of Company X. Company X supplied food outside healthcare settings and it is possible that the patient consumed products from this manufacturer in the community after the initial hospital discharge and before readmission. However, the most likely food exposure was through consuming sandwiches from this manufacturer in hospital. Salads produced by Company X were also contaminated by the strain of *L. monocytogenes* implicated with the clinical case, and at the time of this case, this company supplied, on average, 12 600 salads and sandwiches per day to health care environments. The consumption of salad products has been identified as associated with incidents elsewhere [26, 27]. The characterisation of *L. monocytogenes* isolates from unrelated testing of food or the environment and comparison to isolates from cases of listeriosis was the most common method of initially identifying food vehicles amongst cases in England and Wales during 1981–2015 [3].

It is generally agreed that there is a dose response for human foodborne listeriosis with a greater chance of contracting invasive disease with increasing numbers of *L. monocytogenes* consumed [28]. It has been estimated that 92% of invasive listeriosis cases for all age-gender groups are attributable to doses above 10^5^ cfu per serving. This estimate assumes an average serving size of 50 g, which correspond to an *L. monocytogenes* concentration in ready-to-eat foods of >2000 CFU/g at the time of consumption. This assessment also estimated that the probability of illness varied up to 100 million times between the least to the most susceptible members of the population and that a single *L. monocytogenes* may cause infection [28]. The levels of contamination from products produced from Company X is therefore likely to be low and corresponded to the observation of only one single sporadic case resulting from this food manufacturer. However, the case here consumed sandwiches from this manufacturer on 12 occasions in the weeks prior to the onset of illness, and there may be cumulative effects from multiple exposures. Listeriosis outbreaks described elsewhere have reported unexpected numbers of cases following relatively low levels of contamination after consumption of butter [29] or ice cream [30] which were likely to have been eaten on multiple occasions in hospital or other healthcare settings.

A national outbreak of nine human listeriosis cases occurred in England 2019 which was also associated with pre-prepared sandwiches served in hospitals [11], and was completed unrelated (both by the manufacturer of the sandwiches and the type of *L. monocytogenes*) to the sporadic case described here. Further incidents of listeriosis associated with the consumption of pre-prepared sandwiches served in hospitals in England and Wales have been previously reported [3, 9, 10]. In 2008, an outbreak of 57 cases (24 deaths) occurred in Canada where 72% of the cases were residents of long-term care facilities or hospital inpatients [31]. The Canadian outbreak was associated with the consumption of cold meats and prepared sandwiches, and the meats were produced by a single manufacturer and environmental persistence of the outbreak strain within this meat manufacturing plant was detected. The outbreak resulted in considerable morbidity, mortality and economic loss [31, 32]. Risk factors for these incidents in England and Wales (as well as that in Canada) show many similarities to the sporadic case described here where the exposures were from pre-prepared foods served in hospital where contamination occurred at the point of manufacture. European legislation for foodstuffs in force in England when this incident occurred in 2017 [33] contains microbiological criteria for the presence of *L. monocytogenes* in ready-to-eat foods. Where foods are able to support the growth of *L. monocytogenes*, the regulation requires that *L. monocytogenes* should not exceed 100 cfu/g when products are placed on the market during their shelf life, or if this cannot be demonstrated, there should be absence in a 25 g sample before the food has left the immediate control of the food business operator. For food unable to support the growth of *L. monocytogenes*, the bacterium should not exceed 100 cfu/g in products placed on the market during their shelf life: there are no criteria in this category for foods before they have left the immediate control of the food business operator. Products with a shelf life of <5 days (which usually includes sandwiches) are categorised as unable to support the growth of this bacterium in this regulation [33]. Microbiological guidelines are also available from PHE and the British Sandwich Association. The PHE guidelines for food collected from healthcare environments recommends *L. monocytogenes* not detected in a 25 g samples of ready-to-eat foods [34]. The British Sandwich Association states that for ready-to-eat foods, the ‘aim to be free from detectable *Listeria* (i.e. <20 cfu/g) but accept the EU limit of 100 cfu/g at any point of the products shelf life’ [35].

Methods used to detect *Listeria* in food production environments must be of optimal sensitivity. Company X employed a contract food microbiology laboratory to test samples. Although the laboratory was accredited to the ISO-17025 standard through United Kingdom Accreditation Services (UKAS), it did not detect the contamination detected by the PHE FW&E laboratory. It was not possible to investigate the reason for this discrepancy, although it may be because of test sensitivity. The decisions on using testing laboratories and testing regimes may not be well understood by food manufacturers and made on the basis of cost but not on testing method performance. For food manufacturers to verify their HACCP systems and apply appropriate controls to their manufacturing environment, analytical test methods must be used to detect *L. monocytogenes* in 25 g samples and not just above the 100 cfu/g limit as required in EU legislation [33].

Inspection of Manufacturer X's factory identified problems with drains and a build-up of food debris in the food production environment. Persistent contamination by individual strains provides both the basis for outbreaks as well as indicating a loss of control of the food safety management systems within an individual manufacturing environment. The considerable effort (including testing more than 3000 samples for the presence *Listeria*) was spent by both the public sector and this individual manufacturer to investigate and control *Listeria* contamination in Company X's premises. Since *Listeria* is widespread in the environment [1], successive waves of contamination by different strains of *L. monocytogenes* as well as different *Listeria* species occur. Raw ingredients (including salad products) will inevitably be contaminated from time-to-time, and data in this report show contamination by *L. monocytogenes* other than the incident strain over periods of a few weeks to months. The persistent colonisation by the CC121 strain was analysed by bacterial population dynamics analysis using sequences from more than 120 isolates and estimated to have occurred around 2014 and may coincide with changes within this factory. Similarly, to the contamination by *L. monocytogenes* CC121 in this food chain, there was evidence for colonisation of Company X's food production environment with other *Listeria* species over specific periods although genetic analysis of other *Listeria* species was not carried out. These observations represent long-term colonisation of factory sites with consequential contamination of equipment, food contact surfaces and foods. This study is important in that it provides further strong evidence of the ability of an *L. monocytogenes* clone to persistently affect a food production facility forming a resident microbiota and then the foods produced therein. At the same time, other clones of *Listeria* were transiently present in the facility. This affirms the risk that particular clones have a high degree of fitness for such environments, as seen with the low level of diversity seen in this particular clone over the long sampling period. Mechanisms for the persistence of this bacterium are not well understood and although this may involve resistance to the sanitisers used within a specific environment, other mechanisms may be involved including nutritional exclusion, phage, quorum sensing, genetic advantage, interaction with other parts of the microbiota, etc. Understanding the persistence of *L. monocytogenes* is important for mitigating public health risks to all those involved with the control of *L. monocytogenes* in the food chain (particularly food business operators).

Since the risk factors for listeriosis incidents associated with pre-prepared sandwiches served in hospital show many similarities to those described previously [3, 10], their repeated occurrence despite the production of guidance on reducing the risk of contracting listeriosis in healthcare settings [36] strongly suggests that lessons are not being learnt and opportunities are being missed for prevention. Although there may be problems with temperature control of these products in hospital [10], the most effective intervention will be to prevent contamination at the point of production. There are also opportunities for improvement by the National Health Service to exercise greater control of purchasing based on the specification of products with respect to the absence of *L. monocytogenes*. An independent review of hospital food was reported in 2020 [37] and it is hoped that this will address some of the food safety issues highlighted in the report.

## Data Availability

The datasets used or analysed during this study are available from the corresponding author on reasonable request. Sequence reference numbers appear in supporting data (https://www.ncbi.nlm.nih.gov/bioproject/?term=PRJNA248549). see Supplementary data.

## Supplementary material

For supplementary material accompanying this paper visit http://dx.doi.org/10.1017/S0950268821001916. click here to view supplementary material
